# Effectiveness and feasibility of residential passive heat adaptation interventions on reducing indoor temperature in low-income communities in Africa: a systematic review

**DOI:** 10.1088/2752-5309/ae87bf

**Published:** 2026-07-22

**Authors:** Ritah Nakanjako, Y T Eunice Lo, Ebrahim Behardien, Vuyisile Moyo, Millicent Afi Sitsofe Kwawu, Queen Marekera, Doreen Larkailey Lartey, Mark New, Guy Howard

**Affiliations:** 1 School of Civil, Aerospace, and Design Engineering and Cabot institute for Environment, University of Bristol, Bristol, United Kingdom; 2 Cabot Institute for the Environment, School of Geographical Sciences, and Elizabeth Blackwell Institute for Health Research, University of Bristol, Bristol, United Kingdom; 3 African Climate and Development Initiative, University of Cape Town, Cape Town, South Africa; 4 Institute for Environment and Sanitation Studies, University of Ghana, Accra, Ghana

**Keywords:** indoor temperature, residential passive heat adaptation interventions, FAME framework, low-income communities, Africa

## Abstract

Rising global temperatures have emerged as a critical concern in recent decades and are recognized as one of the biggest threats to human health. In Africa, low-income communities face disproportionate exposure and vulnerability to extreme temperatures due to poorly planned housing structures and limited adaptive capacity. As passive heat adaptation interventions gain traction across the continent, this review evaluates their technical effectiveness and community feasibility within low-income African communities. Using the Joanna Briggs Institute feasibility, appropriateness, meaningfulness, and effectiveness framework, the review examines how building modification interventions have been developed, tested, and implemented, their capacity to improve indoor thermal comfort, and the socio-technical factors influencing their uptake and scalability. The findings indicate that greening systems, house insulation, screened windows, reflective surfaces and window opening have the most potential for widespread and sustainable implementation in low-income communities. In contrast, interventions such as solar chimneys, metal roofs, wind towers, nozzle air funnels, closed eaves, open eaves, thatched roofs, bottle houses, earthbag houses and passive solar houses demonstrate higher context-specific and structural limitations despite their technical effectiveness. The review identifies critical gaps in long-term performance, scalability and community acceptability of technically effective passive heat adaptation interventions, especially in low-income communities. Overall, the study provides an analytical foundation for guiding the selection of effective, contextually appropriate passive heat adaptation interventions and underscores the need for participatory and inclusive implementation approaches to enhance heat resilience in vulnerable African communities.

## 1. Introduction

Rising global temperatures are one of the biggest threats to human health (Campbell *et al* 2018). Although Africa contributes minimally to global greenhouse gas emissions, the continent faces disproportionate impacts from climate-related extremes and is projected to experience intensified adverse effects from rising temperatures (Engdaw *et al* 2022, IPCC 2023). In addition, the continent is experiencing rapid population growth and urbanization, which are driving an increasing demand for housing and expansion of the building sector in both urban and rural areas (Mthiyane *et al* 2022). The pace of this growth has led to poorly planned infrastructure and widespread informal settlements characterized by high density housing, inadequate services, and structural vulnerabilities. The absence of enforced building regulations, performance-based standards, and coherent urban planning frameworks has reinforced poverty and inequality while exacerbating exposure and vulnerability to extreme heat (Hugo and Sonnendecker 2024). This leads to substantial health risks in low-income communities (Ncongwane *et al* 2021, Laue *et al* 2022).

Housing in these contexts is low-income and often informal, typically constructed through autonomy and incremental processes in which affordability is prioritized, often at the expense of durability and thermal performance (Bredenoord 2015, Francisco and Watanabe 2024). This leads to construction of semi-permanent structures made of low-quality materials, creating environments that are highly vulnerable to heat stress (Naicker *et al* 2017). Furthermore, these informal settlements are often ignored within housing policies and initiatives due to their presumed interim status and illegitimacy, resulting in a persistent gap in linking their housing conditions to thermal performance and their lived realities of heat stress exposure (Hugo and Sonnendecker 2024).

Recently, there has been a surge in studies focusing on the heat, health and housing nexus in Africa (Naicker *et al* 2017, Koranteng *et al* 2021, Wilby *et al* 2021, Laue *et al* 2022). These have demonstrated the risks of increasing indoor temperatures induced by different building types within low-income and rural communities. Housing characteristics such as insulation, age of the dwelling, ceiling types, roof types, wall materials, and house orientation have emerged to play a significant role in regulating indoor thermal conditions. However, to combat increasing heat, households often rely on short-term behavioural adaptation practices like hydrating, activity planning, foot immersion, and self-soaking (Adegun and Ayoola 2022, Laue *et al* 2022). Such behaviours are important but insufficient in addressing rising indoor heat and the attendant health risks. Therefore, there’s an increasing interest in passive heat adaptation strategies and innovative housing designs that can improve indoor thermal comfort. Previous studies have tested various approaches such as evaporative cooling, cavity walls, cool roofs, and insulation, demonstrating their potential to reduce heat exposure in diverse African contexts (Anantha Lekshmi and Stalin Jose 2020, Moghayedi *et al* 2022, Boumlik *et al* 2024, Birge *et al* 2025). However, there remains limited research on community feasibility in low-income settings. Existing studies often emphasize technical outcomes without fully addressing affordability, accessibility, scalability or the implications of self-build construction practices that dominate housing in these settings. This review addresses this gap by evaluating the technical effectiveness and community feasibility of building passive heat adaptation interventions in low-income African communities together.

Specifically, this review examines how such interventions have been developed, tested, and implemented, their capacity to improve indoor thermal comfort, and their socio-technical factors influencing their uptake and scalability in low-income communities. Using the Joanna Briggs Institute (JBI) FAME framework (feasibility, appropriateness, meaningfulness, and effectiveness) (Hu and Xu 2024), we provide a comprehensive assessment of resource availability, costs, cultural acceptability, scalability, and community participation in intervention processes. Through this analysis, we aim to present evidence to support identification of the most effective interventions, identify future research priorities and policy response to reduce heat vulnerability in African low-income communities.

## 2. Methods

We undertook a systematic review of published literature using the PICO (population, intervention, comparison, and outcome) eligibility criterion framework described in Tufanaru *et al* (2017). A research protocol was created based on the PRISMA guidelines (Page *et al* 2021).

### 2.1. Search strategy

We searched for papers in Scopus, Web of Science, ProQuest, Compendex (Engineering village), ScienceDirect, and Google Scholar This was done from 6 October 2024 to 11 December 2024. In addition, reference lists and citations of included publications were hand-searched for relevant articles. An initial search strategy was conducted in Scopus and Web of Science to capture relevant search terms and create a comprehensive search strategy for other databases. The search terms are set out in table 1. There was no time restriction placed on records. Only papers in, translated to and with versions of english were included.

**Table 1.** Search terms used to extract records.

Database	Search terms
Scopus	(TITLE-ABS-KEY (heat AND adaptation AND africa AND indoor) OR TITLE-ABS-KEY (africa AND passive AND cool*)), (TITLE-ABS-KEY (passive AND heat* AND adaptation* AND strategies AND africa AND indoor AND temperatures) OR TITLE-ABS-KEY (heat AND adaptation AND to AND increasing AND heat AND in AND africa)), TITLE-ABS-KEY (heat AND stress AND adaptation AND informal AND settlements AND africa), TITLE-ABS-KEY (heat AND stress AND housing AND africa), (TITLE-ABS-KEY (green AND roof* AND in AND africa)), (TITLE-ABS-KEY (cool AND roof* AND in AND africa))
Web of Science (WoS)	ALL = (africa AND passive AND cool, ALL = (heat AND adaptation AND africa AND indoor), ALL = (heat AND adaptation AND to AND increasing AND heat AND in AND Africa, heat AND stress AND adaptation AND informal AND settlements AND africa), ALL = (heat AND stress AND housing AND africa), ALL = (indoor AND temperatures AND house AND africa, green AND roof* AND in AND Africa, cool AND roof* AND in AND Africa))
ProQuest	Passive cooling in Africa, heat AND stress AND adaptation AND informal AND housing AND Africa, cool AND roof* AND in AND Africa
COMPENDEX (engineering village)	((heat AND Adaptation AND Strateg* AND Africa) WN ALL), ((((africa) WN ALL) AND ((passive) WN ALL)) AND ((cool*) WN ALL)), ((heat AND adaptation AND to AND increasing AND heat AND in AND africa) WN ALL), ((heat AND stress AND housing AND africa) WN ALL), ((passive AND cooling AND strategies AND houses AND Africa) WN ALL), ((indoor AND temperatures AND house AND africa) WN ALL), ((Residential AND cooling AND in AND Africa) WN ALL), ((green AND roof* AND in AND africa) WN ALL), ((cool AND roof* AND in AND africa) WN ALL)

### 2.2. Inclusion and exclusion criteria

Population

The review focused solely on residential house modifications in Africa specifically in low-income communities, without considering the population demographics of the people living in the houses during the implementation of interventions.

Articles that had a wider focus but included and provided African evidence were included. Articles that presented results on non-residential buildings were excluded.

Intervention

All interventions related to building modifications that are designed to improve indoor thermal comfort through passive cooling systems were included. These were either implemented by a study or the study monitored existing interventions in isolation or combination and if additional to existing active heat adaptation interventions. Interventions to install active cooling or heating technologies (e.g. air conditioning) were excluded.

Comparison

We looked for reported measures that compared before and after interventions, compared different interventions and those that used case-control designs; where controls were defined as settings or structures without the implemented intervention.

Outcomes

The review targeted two primary outcomes.

1) Intervention results: The review assessed the quantitative outcomes of each intervention in addressing indoor thermal comfort. Additionally, it evaluated whether potential adverse effects associated with implementation were reported.
2) Feasibility assessment of interventions: Using the JBI FAME framework (Hu and Xu 2024), the review conducted a feasibility assessment and recommendation for implementation of interventions in low-income communities in Africa.

In the JBI FAME framework, feasibility (F) refers to the practicality of an intervention in a given context, considering costs, resource availability, and the presence of adequate skills and expertise. Appropriateness (A) assesses cultural acceptability, scalability and adaptability of an intervention across different contexts. Meaningfulness (M) focuses on participants’ autonomy, choice, and shared decision-making, to inform the positive experiences of individuals in response to the intervention and effectiveness (E) evaluates the extent to which interventions achieve their intended outcomes.

The FAME framework establishes two grades of intervention recommendation based on the performance of an intervention (Joanna Briggs Institute 2014). Grade A represents a strong recommendation of intervention where (1) its clear desirable effects outweigh undesirable effects; (2) where there’s evidence of adequate quality supporting its use; (3) there’s benefit or no impact on resource use and (4) values, preferences and experiences have been considered. Grade B represents a weak recommendation of intervention where (1) desirable effects outweigh undesirable effects even when it is unclear; (2) where there is evidence supporting its use, although this may not be of high quality; (3) there is a benefit, no impact or minimal impact on resource use, and (4) values, preferences and experiences may or may not have been considered.

### 2.3. Study selection process

Studies included experimental and quasi-experimental study designs, randomized controlled trials, non-randomized controlled trials, and before and after studies. Analytical observational studies including, case-control studies, and analytical cross-sectional studies were also considered. The review considered those which presented a quantifiable output of the intervention and excluded papers presenting qualitative outputs. Published study protocols were excluded. Studies focusing on use of test chambers or test cells as their experiments were included only if the output or one of the outputs is a direct measurement of the test chambers. Studies solely focused on simulations without direct measurements, or where the data was used for validating simulation models prior to output were excluded. All simulation studies done in isolation of actual field experiments, including those focusing on computer modelling, that did not include field data, field house models or experiments and having unverified empirical evidence were excluded. Lastly, all studies focused on energy reduction using passive strategies without an explicit output on thermal comfort changes after intervention were also excluded.

### 2.4. Critical appraisal

The critical appraisal to evaluate the quality of records retrieved was based on the most appropriate tool for evaluation of each record. The reviewers used the JBI checklist for quasi-experimental studies (non-randomized experimental studies) (JBI 2020) and CASP checklist for case studies (CASP 2024). ‘Yes’ indicates the paper ably answered the question, ‘No’ indicates failure to answer the question, ‘N/A’ indicates the question is not applicable and ‘Unclear’ indicates the paper did not distinctly address the question. The results of the critical appraisals are presented in tables 2 and 3. Evaluation of records was done by six reviewers separately and jointly. Disagreements were reviewed and resolved by three additional reviewers.

**Table 2.** JBI checklist for quasi-experimental studies (non-randomized experimental studies).

JBI Question	Is it clear in the study what is the cause and what is the effect (i.e. there is no confusion about which variable comes first)?	Were the houses included in any comparisons similar?	Were the houses included in any comparisons receiving similar treatment/care, other than the exposure or intervention of interest?	Was there a control group?	Were there multiple measurements of the outcome both pre and post the intervention/ exposure?	Was follow up complete and if not, were differences between houses in terms of their follow up adequately described and analysed?	Were the outcomes of houses included in any comparisons measured in the same way?	Were outcomes measured in a reliable way?	Was appropriate statistical analysis used?
(von Seidlein *et al* 2017)	Yes	No	Yes	Yes	Yes	Yes	Yes	Yes	Yes
(Fitchett *et al* 2020)	Yes	Yes	Yes	Yes	Yes	Yes	Yes	Yes	Yes
(Koranteng *et al* 2022)	Yes	Yes	Yes	Yes	Yes	Yes	Yes	Yes	Yes
(Bradley 2022)	Yes	Yes	Yes	No	Yes	Yes	Yes	Yes	Yes
(Akinwolemiwa *et al* 2018)	Yes	No	Yes	Yes	Yes	Yes	Yes	Yes	Yes
(Carrasco-Tenezaca *et al* 2023)	Yes	Yes	Yes	Yes	Yes	Yes	Yes	Yes	Yes
(Carrasco-Tenezaca *et al* 2021)	Yes	Yes	Yes	Yes	Yes	Yes	Yes	Yes	Yes
(Oluwafeyikemi and Julie 2015)	Yes	Yes	Yes	Yes	Yes	Yes	Yes	Yes	Yes
(Jatta *et al* 2018)	Yes	No	Yes	Yes	Yes	Yes	Yes	Yes	Yes
(Jatta *et al* 2021)	Yes	No	Yes	Yes	Yes	Yes	Yes	Yes	Yes
(Imessad *et al* 2014)	Yes	No	Yes	No	Yes	Yes	Yes	Yes	Yes
(Rincón *et al* 2019)	Yes	No	Yes	Yes	Yes	Yes	Yes	Yes	Yes
(Francisco and Watanabe 2024)	Yes	No	Yes	Yes	Yes	Yes	Yes	Yes	Yes
(Bradley *et al* 2021)	Yes	No	Yes	No	Yes	Yes	Yes	Yes	Yes
(Kimemia *et al* 2020)	Yes	Yes	Yes	Yes	Yes	Yes	Yes	Yes	Yes
(Bouchahm *et al* 2011)	Yes	Yes	Yes	Yes	Yes	Yes	Yes	Yes	Yes
(Olawale-Johnson *et al* 2021)	Yes	Yes	Yes	Yes	Yes	Yes	Yes	Yes	Yes
(Dabaieh *et al* 2021)	Yes	Yes	Yes	Yes	Yes	Yes	Yes	Yes	Yes
(Loggia *et al* 2014)	Yes	Yes	Yes	No	Yes	Yes	Yes	Yes	Yes
(Onyenokporo *et al* 2024)	Yes	No	Yes	Yes	Yes	Yes	Yes	Yes	Yes
(Athmani *et al* 2023)	Yes	Yes	Yes	Yes	Yes	Yes	Yes	Yes	Yes
(Overen *et al* 2019)	Yes	No	Yes	No	Yes	Yes	Yes	Yes	Yes
(Makaka *et al* 2008)	Yes	N/A	N/A	No	Yes	Yes	Yes	Yes	Yes
(Adounkpe *et al* 2014)	Yes	Yes	Yes	Yes	Yes	Yes	Yes	Yes	Yes

**Table 3.** CASP critical appraisal checklist for case studies.

CASP question	Did the study address a clearly focused issue?	Did the authors use an appropriate method to answer their question?	Were the case study buildings recruited in an acceptable way?	Were the existing interventions selected in an acceptable way?	Was the exposure accurately measured to minimise bias?	Aside from the exposure, did the houses have similar characteristics?	Have the authors taken account of the potential confounding factors in the design and/or in their analysis?	Was the existing intervention effect large?	Was the estimate of the existing intervention effect precise?	Do you believe the results?	Can the results be applied to other houses of interest?	Do the results of this study fit with other available evidence?
(Mfamadi *et al* 2024)	Yes	Yes	Yes	Yes	Yes	No	Unclear	Yes	Yes	Yes	Yes	Yes
(Liu *et al* 2022)	Yes	Yes	Yes	Yes	Yes	No	Yes	Yes	Yes	Yes	Yes	Yes
(Adaji *et al* 2019)	Yes	Yes	Yes	Yes	Yes	No	Unclear	Yes	Yes	Yes	Yes	Yes
(Wilby *et al* 2021)	Yes	Yes	Yes	Yes	Yes	No	Yes	No	Yes	Yes	Yes	No
(Ayanlade *et al* 2019)	Yes	Yes	Unclear	Yes	Yes	No	Unclear	Yes	Yes	Yes	Yes	Yes

### 2.5. Data collection and extraction

Records were extracted and managed using EndNote (version 21, 2024). Data on authors, year of publication, geographical focus of the publication, publication type, study design, method, sample size (houses and population), intervention category, intervention comparative factors, metric measure of indoor conditions, season of measurement, intervention outcome, study limitations and recommendations were collected and transferred into an Excel file and coded to facilitate analysis. Before analysis, articles were reviewed by a team of six reviewers separately and jointly. The team conducted a test review of six papers to discuss the inclusion criterion. All papers included were reviewed by the lead author. Second screening was undertaken by five authors. Disagreements were discussed by the lead author and the relevant second screener and where this agreement was not reached, this was resolved through discussions with three other reviewers.

## 3. Results and discussion

### 3.1. Search results

**Figure 1.**
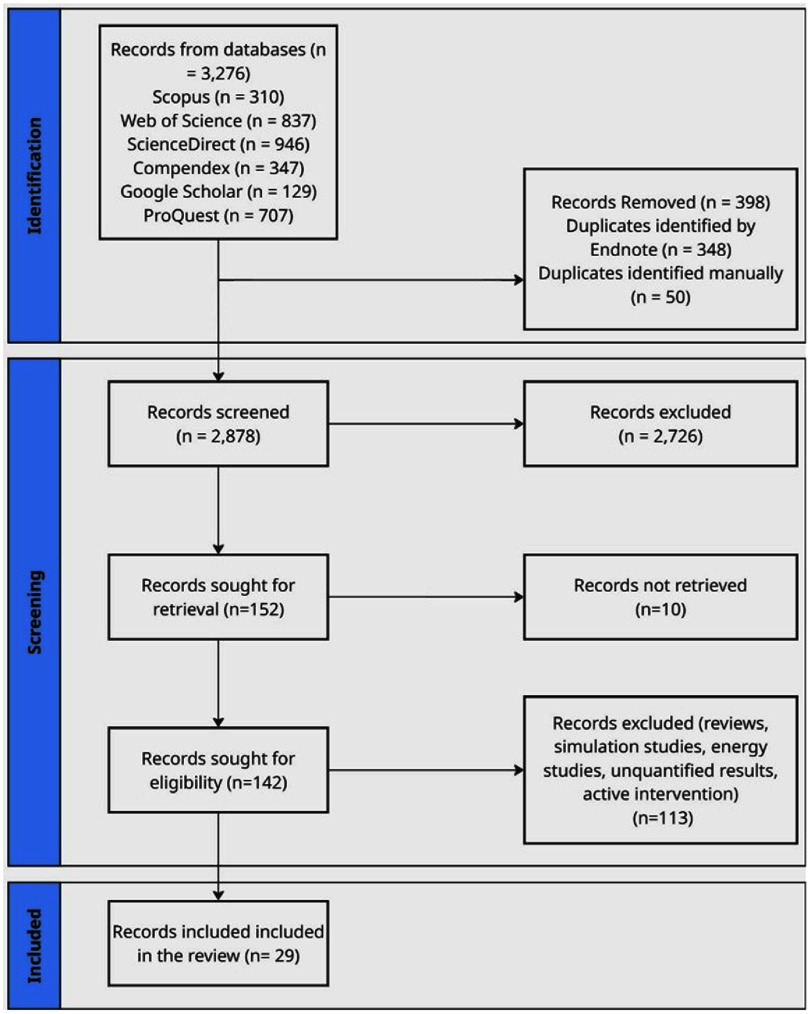
PRISMA framework flow diagram.

A total of 3 276 articles were retrieved. 348 duplicates were identified and removed by Endnote and 50 duplicates were manually removed. After removal of duplicates, the titles of 2 878 articles were screened (figure 1). Using the inclusion and exclusion criteria, 2 726 articles were removed. 152 articles were sought for full text retrieval and screening, and only 142 articles were accessed. During the full text screening, 113 articles did not meet the inclusion criteria. Only 29 articles were eligible for inclusion in this review.

### 3.2. Critical appraisal results

### 3.3. Description of included studies

**Figure 2.**
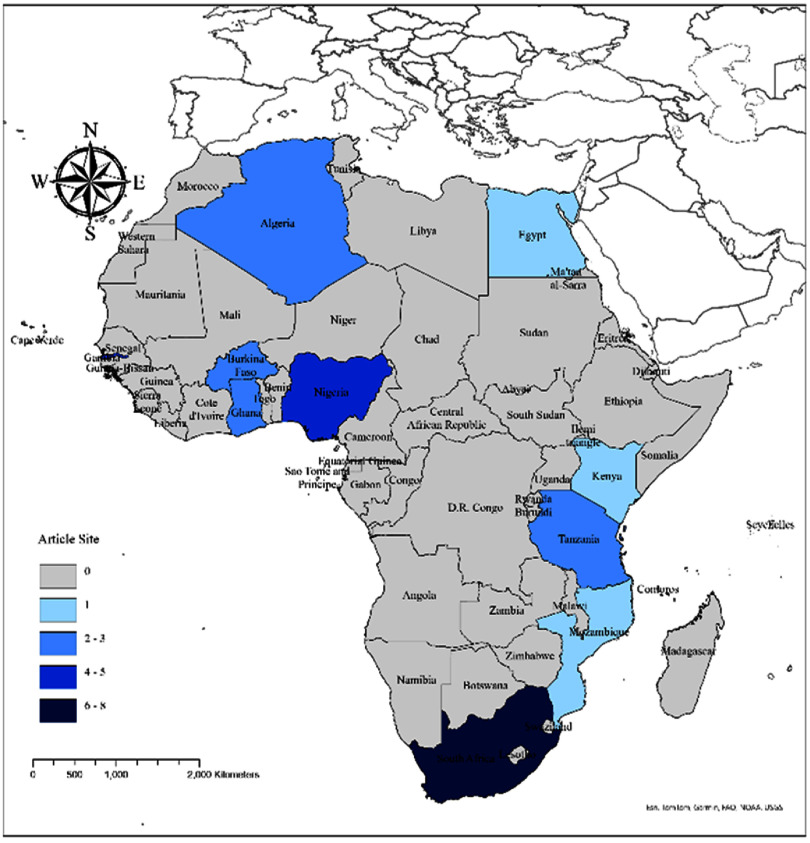
Distribution of articles across Africa.

All articles retrieved were original primary studies. Despite having no time restriction, only papers between 2008 and 2024 met the inclusion criteria. Six articles used an empirical case study design (five case studies and one case study with validated simulations) and twenty-three used an empirical experimental study design (twenty experimental and three experimental with validated simulations). Empirical case studies assessed existing houses without making any structural modifications. Empirical experimental studies either made house modifications, constructed new houses or used testing chambers for assessments. The papers included were studies in nine countries in Africa namely, South Africa (*n* = 8), Nigeria (5), The Gambia (4), Algeria (3), Tanzania (2), Ghana (2), Burkina Faso (2), and Egypt (1), Kenya (1), Mozambique (1) (Figure 2). The studies were conducted in nine different climates: tropical savannah climate, hot desert climate, subtropical highland climate, arid/desert climate, hot semi-arid (steppe) climate, humid subtropical climate, Mediterranean climate, temperate climate, and tropical monsoon climate. Regionally, west Africa had the greatest number of interventions assessed (*n* = 13), followed by Southern Africa (*n* = 8) and East Africa (*n* = 3).

### 3.4. Housing characteristics and population

In reference to community descriptions in this review, some papers defined low-income as communities with majority of the population surviving on less than US$1 per day (Oluwafeyikemi and Julie 2015). Most articles focused on the structural description of houses with limited detail on the populations living in houses. The smallest number of occupants was four in a single house (Makaka *et al* 2008), while the largest included 16 people across five buildings (Onyenokporo *et al* 2024). Several studies, for example Mfamadi *et al* (2024), described numbers of dwelling units investigated but did not specify the occupancy population.

Housing types and experiments varied between studies. Fourteen studies investigated existing house structures in the form of traditional homes, single or double-storied houses, single, double or multiple roomed houses and container buildings largely within low-income communities. Ten studies constructed and investigated controlled human habitable experimental structures or house prototypes, five investigated experimental test chambers, and one had no clear description of housing structure or population.

### 3.5. Interventions categorization

The review identified seven categories of heat adaptation interventions: greening systems, house insulation, house structure, natural ventilation, prototype house development, reflective surfaces, and combined interventions (see table 4 for details). Eleven studies used prototype house development for the purposes of experiments to analyse its effect. Rincón *et al* (2019) explored the indoor thermal difference of an innovative earthbag building, Onyenokporo *et al* (2024) evaluated the thermal comfort of a bottle house construction, while Overen *et al* (2019) and Makaka *et al* (2008) evaluated the performance of low-cost passive solar houses in regulating indoor temperatures. The remaining seven studies constructed prototypes with house modifications on the roofs, windows, doors, cladding materials, house paintings and thermal mass resistance.

**Table 4.** Description of intervention categories.

Intervention	Description	Article
Greening systems	All studies that implemented green roofs and/or vertical greening systems	(Oluwafeyikemi and Julie 2015, Akinwolemiwa *et al* 2018, Fitchett *et al* 2020)
House insulation	Insulation interventions such as fabric insulation, ceiling and roof insulation.	( Adounkpe *et al* 2014, Loggia *et al* 2014, Bradley 2022)
House structure	Studies that did not implement an intervention but used case study buildings and their properties to investigate the effects on indoor thermal comfort conditions. These included house orientation, roof types, window types, ceilings, existing insulators and house shading.	(Ayanlade *et al* 2019, Wilby *et al* 2021, Bradley 2022, Liu *et al* 2022, Mfamadi *et al* 2024)
Natural ventilation	Studies that implemented interventions such as con-shaped shisha funnels, solar chimney, wind towers and assessed natural ventilation.	(Bouchahm *et al* 2011, Adaji *et al* 2019, Dabaieh *et al* 2021, Carrasco-Tenezaca *et al* 2023)
Prototype house development	Studies that reported on constructed case study prototypes incorporating various heat adaptation interventions. For example, constructing earthbag buildings, bottle houses, low-cost passive solar houses, buildings with structures like closed or open eaves, screened windows, and fly ash bricks.	(Makaka *et al* 2008, Imessad *et al* 2014, von Seidlein *et al* 2017, Jatta *et al* 2018, Overen *et al* 2019, Bradley *et al* 2021, Jatta *et al* 2021, Francisco and Watanabe 2024, Onyenokporo *et al* 2024)
Reflective surfaces	Studies focused on cool coatings, cool roofs, reflective surfaces, and assessing different surface albedos using different color paints.	(Adounkpe *et al* 2014, Kimemia *et al* 2020, Carrasco-Tenezaca *et al* 2021, Bradley 2022, Athmani *et al* 2023)
Combined effect intervention	Integrated measurement of different interventions implemented without decoupling the individual contribution of each intervention. The combined effect included solar shading, green roofs and cool paints interventions.	(Olawale-Johnson *et al* 2021)

Greening systems were investigated in four studies. Two studies focused on green roofs (Fitchett *et al* 2020, Koranteng *et al* 2022), while two studies focused on vertical greening (Oluwafeyikemi and Julie 2015, Akinwolemiwa *et al* 2018). Five studies implemented reflective surface interventions using different roofs and wall coatings. Four studies analysed natural ventilation techniques including wind towers, solar chimneys and an innovative cone-shaped shisha funnel system (Dabaieh *et al* 2021). Three studies (Adounkpe *et al* 2014, Loggia *et al* 2014, Bradley 2022) analysed ceiling insulation.

Six studies investigated thermal comfort in existing house structures, examining factors such as roof types, window designs, insulation properties, shading, orientation, and building materials, and only one study (Olawale-Johnson *et al* 2021) focused on the combined effect of multiple passive interventions.

### 3.6. Intervention outcome measures

More than half of the studies (*n* = 18/29) investigated direct indoor air temperature and relative humidity as an outcome effect of interventions. Eight studies used only indoor air temperatures. Bradley (2022) used a CIBSE (chartered institution of building services engineers) overheating criteria to evaluate the effect of interventions. Three studies used derived temperature metrics like operative temperatures (average air temperature and the heat from surrounding surfaces, representing how warm or cool a space actually feels to a person) and apparent temperatures (measure of how hot or cold it feels to a person, considering air temperature and factors like humidity and wind speed). Most of the studies used integrated temperature sensors and data loggers such as thermo-hygrometers for measurements at different time intervals ranging from one minute to one hour. Seasons of measurement varied among studies. Among the studies that recorded seasonal outcomes of interventions, seven studies recorded outcomes for the winter or rainy season, five studies recorded summer or hot season outcomes, and six studies provided outputs for both winter and summer. One study was conducted in spring (Mfamadi *et al* 2024). The longest monitoring period was conducted for three years by Ayanlade *et al* (2019) and the shortest monitoring period was seven days (Imessad *et al* 2014).

### 3.7. FAME analysis

#### 3.7.1. Intervention effectiveness (E)

For a summarized list of intervention impact on indoor thermal conditions, see supplementary material one.

##### 3.7.1.1. Greening systems

**Figure 3.**
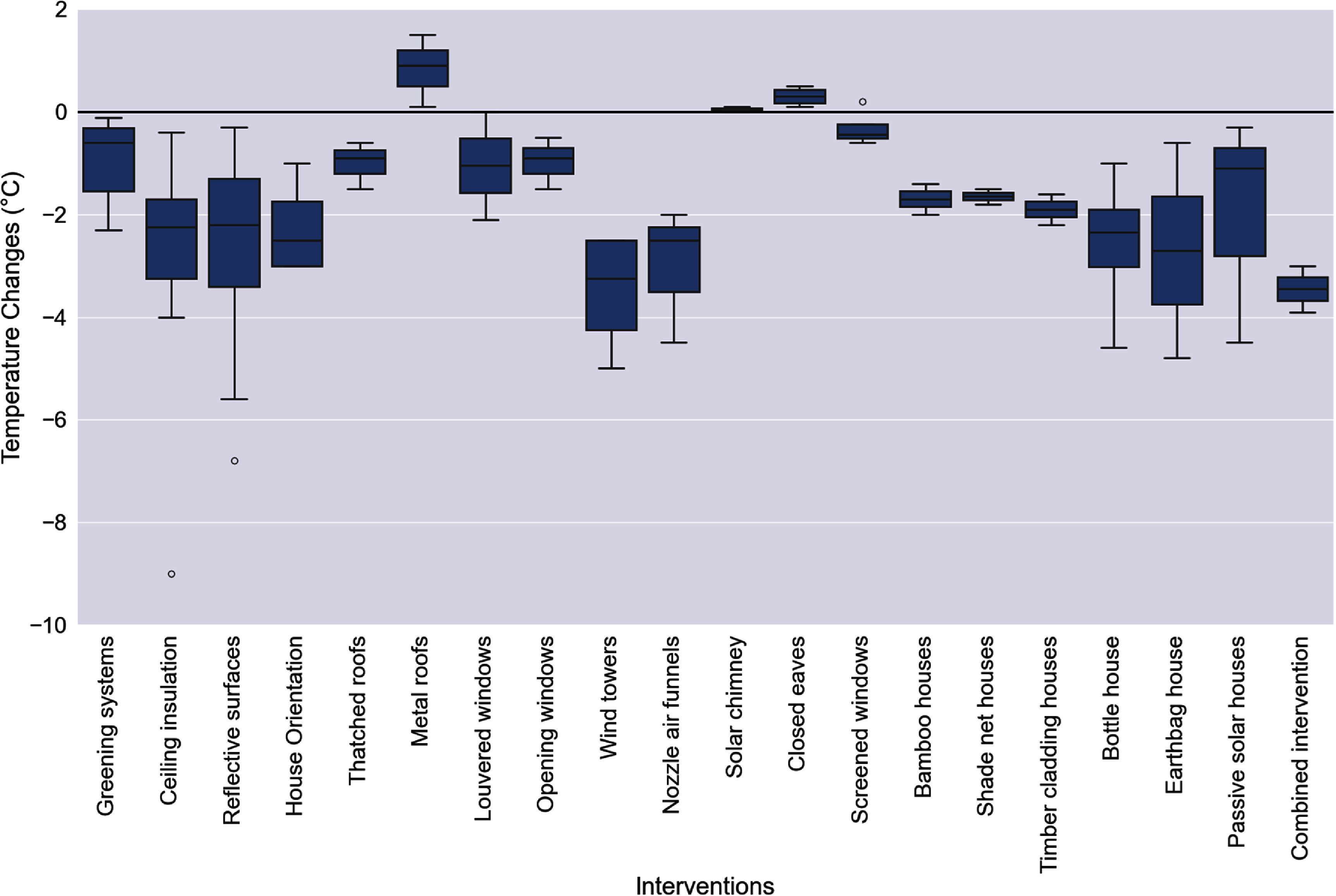
Temperature changes in various interventions.

Greening systems were consistently associated with an average indoor temperature reduction of 2.7 °C, indicating the potential of greening systems as effective passive heat mitigation strategies (see figure 3). In South Africa, Fitchett *et al* (2020), evaluated a vegetated green roof using an indigenous plant species (Plectranthus spp) and reported an average winter indoor temperature reduction of 2.7 °C compared to soil (1.4 °C) and tile roofs (1.3 °C) relative to the roof without a greening system. In Ghana, Koranteng *et al* (2022) assessed green roof systems using Setcreasea and Portulaca vegetation species. They found that Setcreasea achieved greater cooling (by 0.3 °C in the coolest month and 0.4 °C in the hottest month) than Portulaca (0.1 °C and 0.3 °C respectively). In Nigeria, Akinwolemiwa *et al* (2018) and Oluwafeyikemi and Julie (2015) both examined vertical greening systems using various plant holders. Akinwolemiwa *et al* (2018) found that using high-density polyethylene and bamboo plant holders with mixed medicinal, aesthetic and edible plants reduced indoor temperatures by 2.3 °C, while Oluwafeyikemi and Julie (2015) reported reductions of 0.8 °C (bamboo holders) and 1.8 °C (plastic holders).

These findings align with studies (Herath *et al* 2018, Hao *et al* 2020, Rupasinghe and Halwatura 2020) which report similar thermal benefits from greening systems. Notably, Koranteng *et al* (2022) attributed the slightly better performance of Setcreasea over Portulaca to its higher leaf area index (LAI) and enhanced shading effectiveness. Simulation results within the study confirmed that LAI positively correlates with reduced surface and indoor temperatures, consistent with studies by Smith and Roebber (2011) and Zhang *et al* (2015) which highlight the role of LAI in reducing solar radiation influx and absorption on building envelopes. Overall, these results suggest that plants with higher LAI values are more effective in reducing both inner and outer surface temperatures, thus improving indoor and outdoor thermal comfort. However, the performance of greening systems is context-dependent, influenced by outdoor temperature variations, solar radiation exposure, cloud cover, rainfall, and plant resilience (Fitchett *et al* 2020). A comparative analysis by Jamei *et al* (2023) revealed that temperate climates like in some parts of South Africa exhibit a higher magnitude of cooling effectiveness for green system technologies than hot-humid climates like Ghana and Nigeria, likely due to differences in evapotranspiration rates and solar intensity. Nonetheless, the variations in performance across climatic zones were not substantial enough to preclude application in warmer regions. The evidence suggests greening systems can substantially enhance passive heat adaptation, even under varying climatic conditions. The integration of appropriate plant species with higher LAI in greening systems, coupled with local environmental considerations, can optimize cooling performance and improve thermal comfort.

##### 3.7.1.2. House insulation

Ceiling and roof insulation were the predominant insulation types with a maximum reduction of 4 °C–9 °C (figure 3). However, the performance of insulation materials varied widely across climatic contexts, insulation thicknesses, and housing structures. In an experiment in Burkina Faso, Adounkpe *et al* (2014) assessed straw insulation thicknesses of 3 cm and 6 cm, reporting temperature reductions of 12.7 °C and 13.2 °C respectively. While thicker straw had better daytime cooling, it prolonged nighttime heat retention resulting in higher evening indoor temperatures. Thick insulators are associated with longer thermal lags which delays both heat gain and heat loss. This means that during the day, heat flow into the building is minimized, but at night, the inertia of stored heat prevents rapid cooling, potentially compromising thermal comfort (Givoni 2011). However, Adounkpe *et al* (2014) found that although the experiment validated the principle of thermal resistance between thick and thin insulators, there were limitations of extrapolating experimental results to real-world applications.

Similar findings were reported across other contexts. In Ghana, Wilby *et al* (2021) found that insulated rooms were 2.3 °C cooler than uninsulated ones. Insulated metal roofs were 0.8 °C cooler than uninsulated metal roofs, while insulated thatch roofs outperformed insulated metal roofs with a further 0.4 °C reduction. This suggests that the material composition of roof structures play a role in determining overall performance of insulation. In Johannesburg, South Africa, Mfamadi *et al* (2024) reported that apparent temperature differences between insulated and uninsulated container buildings ranged from 2 °C–9 °C. Similarly, in Durban, South Africa, Loggia *et al* (2014) recorded reductions of about 3 °C–4 °C during the hottest period in insulated versus uninsulated houses. In a study in Johannesburg by Bradley (2022), using a CIBSE overheating criteria, found that ceiling insulation reduced indoor temperatures by 2.1 °C during the start of the summer. However, its effect diminished during the peak summer months under extreme temperatures.

These studies indicate that while insulation effectively reduces indoor heat gain during moderate temperature periods, its performance declines under prolonged or extreme heat conditions. The evidence further indicates that while insulation resists external heat gains, it impedes internal heat loss, which may lead to poor performance particularly in areas with high nocturnal temperatures and limited diurnal cooling. Saleh *et al* (2018) note that in persistently hot climates, the mechanisms of heat storage and delayed dissipation can lead to prolonged thermal inertia, narrowing diurnal temperature variations and trapping heat indoors throughout day and night. This pattern suggests that insulation alone may not ensure thermal comfort in tropical or semi-arid climates. Its effectiveness may depend on the integration of adaptive strategies such as cross-ventilation, radiative cooling, and reflective materials (Loggia *et al* 2014). Without these complementary measures, insulation may intensify indoor overheating, especially in poorly ventilated housing.

##### 3.7.1.3. House structure

Studies investigating existing house characteristics and modifications focused on house orientation, house windows, and roof types, revealing variable impacts on indoor thermal performance.

House orientation recorded a maximum reduction of 3 °C, mainly through regulation of solar radiation exposure. In South Africa, Bradley (2022) found that a south-facing vault shaded by an adjacent north vault reduced maximum afternoon summer temperatures by 2 °C, with darker surfaces achieving up to 3 °C of cooling. In a study comparing the thermal conditions of a bedroom and living room of one multi-storied building in Zanzibar, Liu *et al* (2022) found consistently higher temperatures in the living room due to its west-facing orientation which favours absorption of solar radiation compared to the south-facing bedroom. In Algeria, Imessad *et al* (2014), found a reduction in indoor mean temperatures by 1 °C with a south facing wall compared to a west facing wall. Similar findings from Pekdogan and Basaran (2017) and Kontoleon and Eumorfopoulou (2010) demonstrated that north and south facing walls exhibited lower daily heat transfers and temperature ranges compared to those facing east or west across different climates. According to Albatayneh *et al* (2018), the performance of south facing walls is attributed to receiving more diffuse solar radiation in both summer and winter, thereby reducing peak heat gains. Nonetheless, north facing walls outperform south facing walls during winter conditions by allowing winter sun’s radiation and reducing heat loss. These findings indicate that orientation effects may be particularly critical in hot climates, where prolonged exposure to high temperatures can significantly elevate indoor temperatures. However, all studies revealed that surface shading became undesirable in the winter, indicating a seasonal dependent trade-off inherent to orientation-based adaptation strategies.

House window types recorded a maximum reduction of 2 °C primarily due to open area efficiency. In Nigeria, Ayanlade *et al* (2019) found that houses with louvered windows maintained a 2 °C lower indoor temperatures than those with sliding windows during the hottest month. Moreover, louvered windows resulted in 8% lower average indoor relative humidity under both wet and dry seasonal conditions due to their larger opening area. According to Guo *et al* (2024), windows with a smaller opening area such as sliding windows limit air exchange and slow indoor heat dissipation, resulting in heat accumulation during summer daytime hours. These findings underscore the importance of window types and geometry in shaping ventilation efficiency under hot-humid and hot-dry conditions.

Roof types indicated a wide variation in thermal outcomes. In Ghana, Wilby *et al* (2021) found that thatch roofs exhibited mean indoor temperatures 0.6 °C lower than metal roofs, despite the existence of insulation materials in thatch roofs. Similarly, Jatta *et al* (2018) indicated that indoor mean temperatures in metal roof houses were 0.9 °C–1.5 °C higher than thatched roofed houses. The better performance of thatched roofs is attributed to its low thermal conductivity, which slows heat transfer into the building. In contrast, metal sheets, characterized by high thermal conductivity rapidly transmit indoor solar heat, exacerbating overheating during summer and daytime hours even when insulated (Gagliano *et al* 2016, Joshi 2020, Staszczuk and Kuczyński 2021). At night, houses with metal sheets cool rapidly, leading to colder indoor conditions due to the same conductive properties that prevented heat retention during the day (Joshi 2020). These patterns highlight a twofold burden of metal roofs in hot climates, intensifying daytime heat while creating uncomfortable nighttime cold conditions.

##### 3.7.1.4. Natural ventilation

Natural ventilation recorded a reduction in temperature ranging from 0.5 °C to 5 °C. Using two residential case studies in Zanzibar, Liu *et al* (2022) reported lower indoor temperatures in the living room of the single-family house having six operable openings compared to a bedroom with only a south-facing opening. The 0.5 °C temperature difference between living rooms in single-family and multi-storey buildings was attributed to accessibility and usability of window openings, as furniture obstructed airflow in the multi-storied structure. Similarly, in Algeria, Imessad *et al* (2014) reported a 0.9 °C reduction in indoor mean temperatures with open windows compared to closed windows during night ventilation experiments. These results indicate that the effectiveness of natural ventilation is linked to window opening operations, which are determined by occupant behaviour, that varies with demographic characteristics such as age and gender, and indoor occupant activities (Guo *et al* 2024). In addition, under winter conditions, unregulated window opening can increase air change rates, accelerating heat loss and contributing to rapid indoor temperature reduction (Staszczuk and Kuczyński 2021, Guo *et al* 2024). This further highlights the dual performance of natural ventilation under window opening, capable of improving comfort during specific periods and exacerbating discomfort in others.

Comparisons between naturally ventilated and air-conditioned buildings illustrate further limitations. In Nigeria, Adaji *et al* (2019) found that only one naturally ventilated building outperformed an air-conditioned building with a reduction of 1.5 °C in maximum indoor temperatures. On average, air-conditioned houses maintained cooler conditions than naturally ventilated houses. Similar findings are reported by Ai *et al* (2016) and Zhao *et al* (2018). Mechanical ventilation sustains controllable and stable thermal conditions thermal comfort for long periods through rapidly modulating indoor temperatures across seasons (Ai *et al* 2016). Despite the effective thermal performance of mechanical ventilation, the preference for natural ventilation in low-income communities is driven by lower operational costs and reduced energy consumption (Zhao *et al* 2018). However, in hot-humid climates and dense urban areas, natural ventilation can fail. Elevated outdoor temperatures especially within urban heat islands, can easily import heat, exacerbating indoor thermal discomfort (Chen *et al* 2017).

In The Gambia, Carrasco-Tenezaca *et al* (2023) examined the impact of a solar chimney and revealed an slight increase in both day and night indoor temperatures by 0.1 °C, indicating no thermal benefit. This was attributed to use of thicker glass and higher thermal mass materials which retained heat for longer periods and limited chimney induced air movements. Although other studies (Asadi *et al* 2016, Al-Kayiem *et al* 2018, Monghasemi and Vadiee 2018) have demonstrated solar chimney potential in improving ventilation airflow rates, performance is strongly dependent on chimney design parameters like inclination angles, building placement, height, and gap width. To avoid overheating, Carrasco-Tenezaca *et al* (2023) and Zhai *et al* (2011) suggest the use of low heat conductivity materials for construction, readjusting chimney design parameters and complementing the system with other strategies like reflective painting, greening, or ceiling insulation to reduce heat gain.

Wind towers offered better cooling effects. In Algeria, Bouchahm *et al* (2011) assessed the performance of a wind tower and revealed an indoor temperature reduction of 2.5 °C–4 °C (figure 3). Effectiveness was enhanced through evaporative cooling by wetting the wind tower, achieving a 3.5 °C–5 °C reduction. These findings align with prior studies (Sadeghi *et al* 2017, Morales *et al* 2021, Ma *et al* 2024) which highlight wind towers as viable solutions in dry or semi-arid climates where humidity is low and diurnal cooling potential is high. Similarly, Dabaieh *et al* (2021) introduced an innovative natural ventilation system by using cone-shaped shisha funnels embedded into walls. Under dry experiment conditions, a 13-nozzle funnel reduced indoor temperatures by 2.5 °C while a 9-nozzle funnel reduced indoor temperatures by 2 °C. Under wet experiment conditions, the 13-nozzle funnel outperformed a 9-nozzle funnel by reducing indoor temperatures by 4.5 °C. Their effective performance was attributed to using clay materials which have good thermal properties. Additionally, the innovation of different numbers and sizes of air nozzles placed within a wall facilitates the mechanism of creating breathing walls. According to Elgheznawy *et al* (2022), breathing walls are penetrable air layers within the building envelope formulated to create a dynamic air flow, relying on permeable materials to facilitate heat exchange and enhance ventilation.

Collectively, these results reveal that natural ventilation functions effectively only when outdoor conditions, architectural design, and user behaviour align. While ventilation can reduce indoor temperatures by up to 5 °C, its performance deteriorates in hot-humid climates, heat-island zones, or buildings with poor permeability. Moreover, systems such as solar chimneys and wind towers demonstrate considerable potential, but only when material selection and design precision are optimized to local climatic dynamics. These findings suggest some risks of using natural ventilation systems. In climates with extreme temperatures, ventilation may shift from being a cooling mechanism to a heat-delivery system, leading to worse thermal conditions.

##### 3.7.1.5. Reflective surfaces

Reflective surfaces recorded reductions of up to 6.8 °C across the reviewed studies (figure 3). In South African, Bradley (2022) showed that a white-painted roof consistently exhibited the lowest maximum operative temperatures. With closed windows, the white-painted roof was 3.4 °C and 5.6 °C cooler than maroon and dark grey roofs respectively. With open windows, the roof was 1.3 °C and 3.3 °C cooler than maroon and dark grey roofs. Similarly, in The Gambia, Carrasco-Tenezaca *et al* (2021) found that white-painted metal roofs were 1.5 °C lower than bare metal roofs, while red-painted roofs achieved only a 1 °C reduction. Subsequently, Bradley *et al* (2021) also found white-painted vaults reduced temperatures by 0.3 °C more than dark painted vaults. Furthermore, in Johannesburg, Kimemia *et al* (2020) indicated that a coal-coated structure reduced mean maximum temperatures by 4.3 °C and mean minimum temperatures by 2.2 °C. During the hot season in Algeria, Athmani *et al* (2023) showed that a cool reflective white-painted roof reduced indoor temperatures by 6.8 °C, more than a white ceramic tile roof (4.0 °C). Additionally, in Burkina Faso, (Adounkpe *et al* 2014), demonstrated that white-painted aluminium iron sheets were 8.7 °C and 9.25 °C cooler than red and grey tiles respectively. All these findings align with other studies (Joudi *et al* 2013, Garg *et al* 2016, Bamdad 2023, Lu *et al* 2023), confirming the consistent performance of high-reflective surfaces in reducing heat gain.

The thermal behaviour of reflective surfaces is driven by high solar reflectance and low absorptivity, which reduces heat absorption (Costanzo *et al* 2013). This evidence demonstrates that reflective surfaces are effective passive cooling strategies for mitigating daytime heat gain within hot climates which translates directly into improved indoor thermal conditions. However, there’s evident seasonal and diurnal performance trade-offs. Studies such as Carrasco-Tenezaca *et al* (2021), Dias *et al* (2014), Shittu *et al* (2020), and Tang *et al* (2021) observe excessive cooling and an increase in heating demand during nighttime and winter conditions. In climates with large diurnal or seasonal temperature variations.

##### 3.7.1.6. Prototype house developments

Prototype house development recorded a temperature reduction ranging from 0.4 °C to 5 °C, indicating the potential of improving indoor thermal comfort through structural redesigns (figure 3). Two studies evaluated modified duplicates of community houses, while four constructed innovative experimental prototypes incorporating alternative materials (such as bamboo, timber cladding), redesigned windows and doors, modified eaves, adjusted roofs, and increased thermal mass.

In Francisco and Watanabe (2024) found that a constructed prototype with closed eaves, screened windows and well-fitted doors had 0.2 °C higher indoor temperatures. Similarly, Jatta *et al* (2018) indicated an increase of 0.4 °C–0.5 °C in mean temperatures in thatched houses with closed eaves while screened windows reduced mean temperatures by 0.4 °C. These findings align with a study by Knudsen *et al* (2020) who emphasized that the predominant practice of sealing house eaves in malaria prone regions in Africa leads to increased thermal discomfort. Closed eaves and air gaps reduce ventilation rates, increasing both daytime and nocturnal heat retention. Although, Knudsen *et al* (2020) recommends combining open eaves, screened windows and doors with bed nets to balance ventilation and mosquito control, evidence shows persistent challenges in bed-net uptake (Beer *et al* 2012, Von Seidlein *et al* 2012, Nguyen *et al* 2022), complicating the applicability of a combined approach.

Other prototypes demonstrated stronger thermal performance, especially those built with high thermal mass materials. Rincón *et al* (2019) indicated that an innovative earth bag building maintained greater thermal stability than a traditional adobe dwelling, reducing mean temperatures at midnight by 0.6 °C and midday mean temperatures by 4.8 °C. In Onyenokporo *et al* (2024) found that a bottle house prototype outperformed mud and cement houses by a mean indoor temperature reduction of 4.6 °C and 2.2 °C respectively. These innovations suggest that high thermal mass walls act as heat sinks, absorbing daytime heat and gradually releasing it at night, dampening diurnal temperature swings and enhancing indoor thermal stability (Roberts *et al* 2023). Passive solar houses equally showed improved thermal performance. In South Africa, Overen *et al* (2019) developed a passive solar house and without a comparator, found that the entire building remained within the thermal comfort zone (20 °C–24.9 °C) throughout the monitoring period. In addition, the oriented south-facing room outperformed all other rooms in the summer with a maximum difference of up to 1.1 °C. Makaka *et al* (2008) similarly demonstrated that a fly ash brick passive solar house maintained indoor temperatures of 4.1 °C and a relative humidity of 22% lower than outdoor conditions. Several studies (Figueiredo *et al* 2016, Truong and Garvie 2017, Wąs *et al* 2022) have indicated the effectiveness of passive solar houses in enhancing thermal comfort. This is attributed to adopting open plan layouts, house orientation aspects, use of high thermal mass materials, and inclusion of clerestory windows to enable effective thermal regulation.

Constructing prototype houses was the predominant research approach in this review. Despite the great thermal performance, this approach raises critical questions about application to the existing housing stock within low-income communities. Most prototypes assessed were either new constructed experimental models or modified duplications that have limited resemblance to the structural realities of informal or incremental building practices common in low-income communities of Africa. This reliance suggests that current housing infrastructure may be inadequate or poorly constructed to support retrofit interventions, thereby necessitating a focus on whole-house prototype interventions. On the other hand, it positions prototype-based approaches as a pragmatic and potentially necessary pathway to ensure effective and sustainable heat adaptation solutions. They offer proof-of-concept demonstrations of what sustainable designs can achieve without constraints of existing building materials and community construction preferences. This review therefore suggests that while prototype houses are valuable for technical innovation, their real-world relevance depends on addressing the infrastructural barriers that shape the lived realities of low-income communities. Without pathways for incremental adaptation, thermally effective prototypes risk remaining conceptual rather than actionable. Ultimately, prototype development must be understood as part of a broader strategy requiring context-sensitive, socially embedded, and economically viable approaches to heat adaptation in low-income housing.

##### 3.7.1.7. Combined effect intervention

Only one study evaluated the combined effects of solar shading, green roofs and cool paints. Olawale-Johnson *et al* (2021) conducted an experiment using two mild steel sheets test boxes (1 m × 1 m base and 2 m height). The experiment box incorporated reflective coated roof, a 30° roof tilt for solar shading and a vegetated roof while the control box remained unmodified. The combined intervention indicated a maximum indoor temperature reduction of 3.9 °C relative to the control box. Although the study does not disaggregate the effect of each intervention, the findings suggest that combined interventions can yield thermal benefits, highlighting the potential of integrated passive design approaches in improving indoor thermal environments.

It is important to note that, except for prototype house development and studies examining inherent house structure characteristics, most interventions identified in this review were implemented as retrofits to existing informal housing. These retrofit approaches including greening systems, reflective surfaces, insulation, natural ventilation modifications or combined are more reflective of practical, short-term adaptation strategies applicable to current low-income communities. In contrast, prototype house developments represent novel constructions, which operate under different thermodynamic and socio-economic conditions and may have more limited immediate applicability for large-scale implementation in existing informal housing contexts. These aspects are further explored in sections 3.7.2–3.7.4.

#### 3.7.2. Intervention feasibility (F)

Feasibility, as defined within the FAME framework, was evaluated based on the practicality of an intervention, costs, resource availability, and the presence of adequate skills and expertise. Only eight studies reported on this component. For reflective roofs, Carrasco-Tenezaca *et al* (2021) compared costs between The Gambia and South Africa. In The Gambia, Carrasco-Tenezaca *et al* (2021) found that a bare metal roof cost US$42, a galvanized corrugated roof with white paint cost US$72 while a galvanized corrugated roof with white reflective coating cost US$136. The same materials in South Africa ranged from US$154 to US$224. This implies the materials are more expensive in South Africa. This could partly be influenced by national price levels and household economic factors which determine commodity prices (Kira 2013, Nakamura *et al* 2020). For a solar chimney experiment, Carrasco-Tenezaca *et al* (2023) estimated a cost of US$517 construction cost, excluding labour. Moreover, the technical expertise required for installation meant that community participation was minimal. In contrast, interventions utilizing local materials showed lower implementation costs. Akinwolemiwa *et al* (2018) estimated a cost of US$373 and US$271 for green wall prototypes using bamboo and timber respectively. Furthermore, the study indicated that materials could be sourced from local markets and installed through co-implementation with participants. However, specialized labor for bamboo shearing increased labor and transport costs, raising concerns that increased demand could escalate prices over time.

Jatta *et al* (2018) reported house modification costs in The Gambia ranging from US$460 to US$589 for thatched roofs and US$540 to US$672 for metal roofs, though labour expertise was not clearly documented. In Mozambique, Francisco and Watanabe (2024) showed that small upgrades such as adding screened windows raised costs from US$105 for a traditional house to US$120, demonstrating the cumulative cost of modern construction aesthetics and materials. More extensive prototype constructions showed higher implementation costs. In Tanzania, von Seidlein *et al* (2017) reported that a traditional house cost US$4 231, whereas incorporating screened windows increased costs by 32% (US$1 989). Double-storied shade-net houses (US$4 661) and timber-clad houses (US$6 125) were even more costly. While participants preferred timber-clad designs, concerns about timber scarcity, deforestation-driven price inflation, and long-term material availability undermined feasibility of these prototypes. Community members also expressed concerns regarding the affordability of adopting such prototype houses. By contrast, low-cost bottle house prototypes in Nigeria (Onyenokporo *et al* 2024) cost US$4 725 and used approximately10 000 PET bottles collected locally. This suggests co-designed, and community-sourced interventions can offset material and construction costs.

No detailed implementation costs were reported for wind towers, air-nozzles, passive solar house or house insulations. Prototype house developments emerged as the most expensive implementation category.

Studies (Sarwar and Alsaggaf 2020, Juffle and Rahman 2023) report high construction and material costs as major barriers to adopting heat adaptation strategies, especially house prototypes, particularly in low-income communities. While some studies (Akinwolemiwa *et al* 2018, Teotónio *et al* 2020) suggest that households may exhibit greater willingness to pay when the long-term benefits of interventions are understood, other studies such as (Liu *et al* 2023) show that willingness to adopt adaptation strategies often depends on external financing, government support, or cost-sharing models, rather than self-funded initiatives. This reliance on external actors may limit the sustainability, and ownership of interventions in low-income communities. Nonetheless, despite the disparities, the implementation costs may not reliably inform household-level affordability especially in low-income communities. Some studies (Goudge *et al* 2009) continue to indicate that individual-level constraints like low personal income, unemployment, weak social networks, and limited awareness exert a stronger influence on adoption decisions of households to invest in interventions. Furthermore, substantial variations in purchasing power parity (PPP) across African countries indicate unequal household capacity to afford heat adaptation interventions. For instance, by 2024, South Africa’s PPP (∼$15400) was considerably higher than that of Nigeria (∼$9000), Tanzania (∼$4200), and The Gambia (∼$3400) (World Bank 2024). These disparities suggest that households in stronger economies are more likely to have greater household spending capacity and better ability to afford housing improvements or materials. In addition, interventions considered low-cost in relatively stronger economies may remain financially inaccessible in weaker economies. Therefore, the economic feasibility of heat adaptation strategies is highly context dependent. Cost is not just an economic variable but also a structural determinant of heat adaptation interventions within low-income communities. Addressing implementation cost challenges may require more than lowering prices. It may involve decisions on context-appropriate designs, strengthening local supply of materials and creating community-based implementation processes to reduce community financial burdens.

#### 3.7.3. Interventions appropriateness (A)

Appropriateness, as defined within the FAME framework, was assessed based on cultural acceptability, scalability and adaptability of an intervention. Very few studies reported on this component.

For greening systems, despite climatic dependency on technical effectiveness, there generally considered scalable and adaptable, capable of being implemented as vertical green walls or green roofs (Fitchett *et al* 2020, Koranteng *et al* 2022). Acceptability was enhanced by the use of locally available materials, perceived visual improvements (Akinwolemiwa *et al* 2018, Fitchett *et al* 2020), and the potential for co-benefits such as stormwater management, improved well-being, and urban agriculture (Sarwar and Alsaggaf 2020, Teotónio *et al* 2020, Joshi and Teller 2021). Notably, the incorporation of edible and medicinal plants increased community willingness to adopt these systems by providing commercial value and health benefits (Akinwolemiwa *et al* 2018). However, long-term adoption remains limited by maintenance requirements, concerns about roof loading and, critically, landlord-tenant dynamics within low-income communities. According to Akinwolemiwa *et al* (2018), in rental-dominated low-income communities, households often lack decision-making power to authorize structural modifications. These constraints indicate that while greening systems may be acceptable, their scalability is strongly moderated by tenure insecurity and the demands associated with plant maintenance.

Solar chimneys and wind towers exhibited significant technical potential but low community appropriateness. Both Bouchahm *et al* (2011) and Carrasco-Tenezaca *et al* (2023) reported that effective implementation requires technical expertise to configure parameters such as chimney size, opening dimensions, angulation, gap-to-height ratios, and number of inlets to ensure optimal performance. Studies (Sadeghi *et al* 2017, Hassan 2023, Ma *et al* 2024) further highlight similar constraints, indicating that the increasing need to integrate interventions like wind towers with modern intelligent systems or renewable technologies increases implementation complexity. From a FAME perspective, these technologies show high technical necessity and limited community-led uptake and scaling.

Within the prototype development approach, acceptability revealed socio-cultural trade-offs. In malaria-prone settings, such as The Gambia and Mozambique, communities strongly preferred closed eaves despite their demonstrated role in increasing indoor temperatures (Jatta *et al* 2018, Francisco and Watanabe 2024). This preference reflects the dominance of vector-control priorities and structural protection over thermal comfort. Efforts to promote open eaves combined with bed nets have struggled due to widespread dissatisfaction with bed-nets, deeming them thermally uncomfortable, unfavourable in shared sleeping spaces, and perceived inconvenience (Beer *et al* 2012, Von Seidlein *et al* 2012, Pooseesod *et al* 2021, Nguyen *et al* 2022). Similarly, communities consistently favoured metal roofing sheets over traditional materials, despite evidence that metal roofs elevate indoor temperatures. This preference reflects cultural shifts toward modern housing rather than thermal comfort considerations (Jatta *et al* 2018, Ondiba *et al* 2018). Combined preferences for closed eaves and metal roofs therefore illustrate intervention challenges between technically effective approaches and community-valued aesthetics and perceptions. Alternative modifications such as screened doors and windows were widely accepted (Francisco and Watanabe 2024), suggesting that interventions aligning with existing cultural and community expectations may provide more scalable pathways to both improved thermal performance and mosquito control.

Co-implementation approaches improved acceptability of bottle and earthbag houses (Oyinlola *et al* 2018, Rincón *et al* 2019). However, significant barriers remain. Bottle houses yielded community dissatisfaction for their aesthetic appearance, which conflicted with cultural shifts to modern housing (Tusting *et al* 2019). Scalability is also challenged by the large quantities of PET bottles required. Although feasible for a single prototype unit, sourcing thousands of bottles becomes impractical. In addition, Oyinlola *et al* (2018) highlights that bottles being associated with a particular segment of the circular economy; reusing bottles for storing things like cooking oil, kerosene or portable water further undermines the uptake of bottle houses. Earthbag houses face environmental and regulatory obstacles. Their construction requires large volumes of specific soil types (Rincón *et al* 2019), and also raises concerns about soil degradation, loss of agricultural fertility, and increased mosquito breeding sites in excavation pits (Hashemi *et al* 2015). Additional barriers include uncertainty regarding durability, building code compliance, and community scepticism toward non-conventional materials (Mahmoud *et al* 2013, Kumar Sharma *et al* 2021). For passive solar houses, while technically effective, require precision in orientation, thermal mass selection, and construction expertise, limiting their appropriateness for informal or self-built housing (Makaka *et al* 2012).

Reflective surfaces showed high acceptability and scalability with minimal cultural constraints. The concern on visibility of dirt and dust on light-coloured roofs as highlighted Carrasco-Tenezaca *et al* (2021) can be mitigated with low-cost cleaning strategies like simple washing with dish detergents, natural removal by wind and rainfall, and professional roof cleaning (Saber *et al* 2021). Cultural considerations, such as potential associations of white colour with mourning which may limit uptake and scalability (Carrasco-Tenezaca *et al* 2021) is grounds for further research.

Thermal insulation presented no major cultural acceptability barriers. Studies such as (Mfamadi *et al* 2024) reported community initiatives of installing insulation materials within their houses for thermal comfort indicating a strong possibility of acceptance. However, thermal insulation appropriateness in hot climates remains debated and hinged on integration with complementary strategies (Loggia *et al* 2014, Melo *et al* 2015, Piselli *et al* 2019, Aggarwal and Molleti 2024).

Generally, these findings indicate a broader perspective that heat adaptation interventions in low-income communities will require integrating technical performance with local meanings of cultural acceptability, and modernity aspirations, ensuring that climate adaptation strategies are compatible with both environmental conditions and socio-cultural realities.

#### 3.7.4. Intervention meaningfulness (M)

Meaningfulness, as defined within the FAME framework, was evaluated based on the extent communities engaged in the decision-making, design, and implementation of interventions. Across the reviewed papers, relatively few studies explicitly addressed this component, indicating a broader gap in participatory integration within heat adaptation interventions within low-income communities. Vertical greening interventions demonstrated the strongest incorporation of community participation. Akinwolemiwa *et al* (2018) used a co-design and co-implementation approach, engaging participants in sketching prototype designs, preparation and sourcing of materials and assembling of equipment. The process was conducted in local languages, facilitating understanding and reinforcing community ownership. Both Akinwolemiwa *et al* (2018) and Oluwafeyikemi and Julie (2015) highlight that positioning designs with community knowledge and preferences improved willingness to adopt and maintain these systems especially within low-income communities where external interventions are often viewed with skepticism.

In The Gambia, Francisco and Watanabe (2024) worked with local builders without formal training, recognizing community autonomy in home construction and supporting local capacity development. Onyenokporo *et al* (2024) incorporated focus groups and interviews into the design and construction of a bottle-house prototype. This approach fostered participant influence in co-designs and decision making which may enhance valued house outcomes. Comparable participatory approaches in constructing the earthbag house, were observed by Rincón *et al* (2019), further illustrating that co-implementation strengthens community engagement and promotes co-ownership of innovative housing models.

In contrast, passive solar houses and technical complex systems such as solar chimneys, nozzle air funnels, and wind towers reported minimal or no community involvement during design or implementation. Their reliance on specialized expertise limits opportunities for participatory decision-making, raising questions about long-term local ownership and viability. Similarly, although reflective surface interventions are technically simple and potentially participatory, reviewed studies did not report community engagement during implementation. Nonetheless, emerging evidence (Bunker *et al* 2024, Deglon *et al* 2025) suggests that reflective surfaces are well suited for co-deployment, involving community groups in application, maintenance, and monitoring, which may enhance meaningfulness and sustainability.

Overall, the limited reporting on meaningfulness across the review indicates a critical gap. Designing interventions that are not only technically viable but also socially meaningful requires embedding participatory processes, and co-implementation within climate adaptation strategies. Without such integration, interventions risk low uptake particularly in resource-constrained and culturally diverse communities.

## 4. Recommendations and implications

### 4.1. FAME grade of intervention suited to low-income communities

Table 5 summarizes the potential of heat adaptation interventions in low-income communities. The evidence indicated that interventions vary not only in thermal performance but also in their social appropriateness, implementation, and potential for long-term community uptake.

**Table 5.** Grade recommendation of interventions.

Grade category	Interventions
Grade A (strong recommendation)	Greening systems, house insulation, screened windows, reflective surfaces and window opening
Grade B (weak recommendation)	Solar chimneys, wind towers, nozzle air funnels, open eaves, bottle houses, earthbag houses and passive solar houses

Greening systems, house insulation, screened windows, reflective surfaces, and window opening are recommended as Grade A interventions. These interventions’ collective demonstration of low technical complexity and compatibility with community practices make them highly suitable for widespread implementation in low-income communities.

Solar chimneys, metal roofs, wind towers, nozzle air funnels, closed eaves, open eaves, thatched roofs, bottles, earthbag and passive solar houses are recommended as Grade B interventions. These interventions are most appropriate under specific conditions with supportive implementation structures, stronger material supply chains, or targeted community partnerships. They may serve as long-term solutions rather than immediate, scalable options. However, interventions such as solar chimneys, metal roofs, and closed eaves, which were associated with increased indoor temperatures, are not recommended as standalone solutions by this review. Their adoption should only be considered where additional measures to reduce indoor heat are incorporated.

This review indicates the value of applying the FAME framework originally implemented within medical and clinical practice for evidence implementation through the JBI evidence-based healthcare model (Jordan *et al* 2022). FAME’s multidimensional lens facilitates a holistic understanding of intervention performance by examining not only what works but also for whom, under what conditions, and with what implications for long-term uptake. Evidence from implementation research shows that FAME supports stakeholder engagement, helps identify barriers, and strengthens pathways for innovation sustainability (Hu and Xu 2024). Despite its proven utility in medical and public health domains, FAME remains underutilized in climate adaptation implementations. This review therefore provides justification for integrating FAME into future heat adaptation planning and research.

### 4.2. Implications for health, research, policy and practice

#### 4.2.1. Health implications

While the reviewed studies primarily report reductions in indoor air or operative temperatures, these changes have important implications on human health that extend beyond heat stress. Indoor temperatures exceeding 26 °C–28 °C are associated with sleep disturbance, reduced cognitive performance, increased cardiovascular strain, effects on diabetes management, core schizophrenia and dementia symptoms particularly among vulnerable populations such as children, older adults, and those with pre-existing conditions (Tham *et al* 2019, Edwards *et al* 2025). Therefore, interventions that increased temperatures (such as solar chimneys, closed eaves) may exacerbate health risks associated with cardiovascular strains by prolonging exposure to elevated indoor heat, particularly in already heat-stressed urban environments.

Nonetheless, several interventions identified in this review such as reflective surfaces (up to 6.8 °C reduction), insulation (up to 4 °C–9 °C), and greening systems (2.7 °C), demonstrate the potential to shift indoor environments closer to physiologically tolerable thresholds. For example, reductions of 2 °C–3 °C can significantly improve sleep quality by enabling nighttime cooling below critical thresholds (Tham *et al* 2019), while larger reductions may reduce risks of dehydration, heat exhaustion, and elevated heart rate associated with sustained exposure to high indoor temperatures (Kenny *et al* 2024). However, the effectiveness of these interventions must be considered within the broader context of diurnal and seasonal temperature variability. For instance, insulation and high thermal mass materials may reduce daytime heat exposure but can also retain heat at night leading to elevated nighttime temperatures (Jannat *et al* 2020, Roberts *et al* 2023). Therefore, in communities with poorly ventilated houses, there’s higher potential of prolonged physiological stress, disrupted sleep patterns and impaired thermoregulation. Similarly, natural ventilation strategies may fail under high outdoor temperatures or urban heat island conditions, inadvertently increasing indoor heat exposure. These findings highlight the need to evaluate passive cooling strategies not only in terms of thermal performance but also in relation to their capacity to reduce adverse health outcomes.

#### 4.2.2. Policy and practice implications

Building modification for passive heat adaptation interventions in Africa aim to ensure indoor temperature reduction, especially within heat exposed and vulnerable communities. This review indicates that community involvement enhances acceptability and long-term scalability of these interventions especially in low-income communities. Policymakers should therefore institutionalize participatory design processes that support co-design and co-implementation frameworks within enactment policies. Given the recurring trade-offs between thermal comfort and health priorities, such as vector control, policy frameworks are required to adopt integrated designs that simultaneously address heat stress, malaria prevention and structure resilience. This integration could be operationalized through building codes and design guidelines that promote combined interventions.

Cost remains a major constraint to uptake, especially for interventions requiring specialized materials, technical expertise and new house redevelopments. This challenge is further compounded in low-income communities where most residents are tenants and landlords often have limited financial motivation to invest in cooling retrofits. Consequently, tenants are either forced to rely on temporary coping strategies or continue enduring prolonged exposure to indoor heat conditions and associated physiological stress. However, encouraging landlords to implement retrofits may also generate unintended consequences, including increased rental costs or tenant displacement as property values rise. These dynamics reflect broader structural inequalities and insecure housing conditions that limit tenants’ ability to influence housing improvements within low-income communities. To address these barriers, governments and development partners should adopt and enhance financial support mechanisms such as targeted subsidies, cost-sharing schemes, and local material market price regulations to promote equitable access.

#### 4.2.3. Research implications

Under the FAME lens, cultural preferences, health risk perceptions, housing aspirations, and maintenance burdens indicate that implementation of interventions is not just reliant on thermal performance metrics. The review underscores substantial research gaps which need to be addressed, particularly in feasibility, adaptability, and meaningfulness beyond technical performance. Most existing studies relied on small-scale assessments through test boxes, small sample sizes and single construction prototypes or short-term thermal assessments. Future research should therefore adopt larger-scale, community-based experimental designs, longitudinal monitoring of thermal performance, and behavioural adaptation. Further investigation is needed to assess the scalability and contextual adaptability of interventions across diverse climatic zones and housing typologies within various communities. Interdisciplinary collaborations between architects, public health experts, and social scientists can facilitate research evaluations that explore financial feasibility models, and socio-cultural factors to identify sustainable pathways for scaling up interventions in low-income settings. Finally, the FAME framework provides a comprehensive approach that assesses both qualitative and quantitative effectiveness of interventions. This review suggests that future studies implementing housing and climate adaptation interventions should incorporate this framework especially within low-income communities where autonomy in house construction remains predominant.

## 5. Limitations and conclusion

### 5.1. Limitations

The available studies were mainly concentrated in the South, East and Western parts of Africa. This limited the review to a few African climate contexts which may fail to adequately address the effectiveness of similar interventions within other countries in Central and North Africa and yet, these areas are known to experience persistent high temperatures (Varela *et al* 2020, Fotso-Nguemo *et al* 2022, Ntoumos *et al* 2022). In addition, a potential limitation of this study lies in the search strategy, which relied on the inclusion of the term ‘Africa’ within titles, abstracts, or full texts. Several records identified through the search included review papers from which additional individual studies were screened and retrieved, increasing the likelihood that most relevant studies were captured. Furthermore, a verification check using selected countries (like Nigeria, South Africa, Algeria, Tanzania) where most studies have been conducted indicated that no additional eligible papers were missed. Although this approach was intended to capture studies conducted across the continent, it may have excluded relevant studies focusing on individual African countries that did not explicitly reference ‘Africa’ in their text.

The application of the FAME framework to assess feasibility was constrained by article inconsistent methodological details and reporting. Information such as intervention costs, resource requirements, and skill levels for implementation was frequently missing. Moreover, evidence on cultural acceptability, scalability, and adaptability of interventions was limited. Few studies examined participants’ experiences or reported on community engagement during implementation. These variations in study samples, methodologies, and reporting reduced the extent to which the review could comprehensively use FAME to evaluate feasibility and limited the comparison of findings across contexts.

### 5.2. Conclusion

This review synthesized evidence on seven categories of passive heat adaptation interventions, to evaluate their potential for enhancing thermal comfort and applicability in low-income African communities. Most studies in this review used a prototype house approach through constructing modified community houses and introducing new house innovations. This approach achieved a maximum indoor temperature reduction of about 5 °C. Other strategies including ceiling insulation, natural ventilation, reflective surfaces and greening systems demonstrated substantial average cooling effects ranging from 2.7 °C to 5.6 °C. In contrast, interventions such as metal roofs, closed eaves, and solar chimneys were found to exacerbate indoor temperatures, with increases of up to 1.5 °C. Altogether, intervention performance varied depending on climate, seasonality, time of day, and construction materials, underscoring the context-specific nature and application of interventions.

Despite the majority of Grade B (weak recommendation) interventions (wind towers, nozzle air funnels, open eaves, bottle house, earthbag house and passive solar house) exhibiting higher indoor cooling performances, this review recommends other interventions (greening systems, house insulation, screened windows, reflective surfaces, and window opening) as Grade A (strong recommendation), as they have the greatest potential for both effectiveness and feasibility in low-income communities. This was mainly due to their affordability, acceptability, and scalability in addition to their acceptable technical performance.

Critical knowledge gaps remain regarding the long-term performance, scalability and community acceptability of technically effective interventions, especially in low-income communities. Researchers should prioritize tackling underlying issues that hinder uptake and scalability through using comprehensive research frameworks like FAME and enhancing interdisciplinary collaborations to identify sustainable pathways. The findings of this review therefore provide a foundation for guiding the selection of effective, contextually relevant passive heat adaptation strategies and emphasize the importance of co-designed approaches within vulnerable populations facing climate change impacts.

## Data Availability

The review used secondary data extracted from the articles which have been appropriately cited. Supplementary Material available at: https://doi.org/10.1088/2752-5309/ae87bf/data1.
